# Mechanical circulatory support and intravascular lithotripsy in high-risk patients undergoing percutaneous coronary intervention and transcatheter aortic valve replacement: a case series

**DOI:** 10.1093/ehjcr/ytab498

**Published:** 2021-12-07

**Authors:** Alfredo Marchese, Giuseppe Tarantini, Antonio Tito, Vito Margari, Fabrizio Resta, Ilir Dhojniku, Domenico Paparella, Giuseppe Speziale

**Affiliations:** 1 Ospedale Santa Maria, GVM Care & Research, Via Antonio De Ferrariis 22, 70124 Bari, Italy; 2 Department of Cardiac, Thoracic and Vascular Sciences, University of Padua, Via Giustiniani, 2. 35128 Padova, Italy

**Keywords:** Extracorporeal membrane oxygenation, Intravascular lithoplasty, Mechanical circulatory support, Transcatheter aortic valve replacement, Intra-aortic balloon pump, Case series, 3.1 Coronary artery disease, 4.2 Aortic stenosis, 6.2 Heart failure with reduced ejection fraction

## Abstract

**Background:**

Patients undergoing transcatheter aortic valve replacement (TAVR) usually have multiple comorbidities, such as severely impaired left ventricular function (LVF) and heavily calcified coronary lesions. When they undergo pre-TAVR high-risk percutaneous coronary interventions (HR-PCIs) for severely calcified left main (LM) lesions, potential life-threatening intra-procedural complications associated with the different techniques available to treat calcified lesions can arise. In this setting, mechanical circulatory support proves its usefulness. However, the choice of device can be troublesome.

**Case summary:**

We report two clinical scenarios of intravascular lithotripsy (IVL) for the treatment of heavily calcified LM coronary lesions, wherein peripheral veno-arterial extracorporeal membrane oxygenation (VA-ECMO), alone or combined with an intra-aortic balloon pump (IABP), were used as an upfront strategy to support the procedure. The use of these techniques was particularly effective during multi-vessel HR-PCIs and TAVR, and no complications occurred, which suggested their safety.

**Discussion:**

These cases provide multiple insights into the strategy of using IVL + VA-ECMO, alone or with IABP, to treat heavily calcified LM coronary lesions in patients with severely compromised LVF undergoing TAVR. IVL safely and effectively overcame shortcomings related to other plaque ablation techniques, and VA-ECMO proved to be effective when facing the combination of high-risk coronary and valve interventions.

Learning points

- Intravascular lithotripsy proved to be safe and useful for overcoming shortcomings related to other aggressive plaque debulking techniques used in the treatment of heavily calcified coronary lesions.
- Veno-arterial extracorporeal membrane oxygenation, alone or combined with intra-aortic balloon pump, could be used to avoid ventricular decompensation in the setting of a complex pre-transcatheter aortic valve replacement high-risk percutaneous coronary intervention.

## Introduction

Transcatheter aortic valve replacement (TAVR) has emerged as a therapeutic option for severe aortic stenosis (AS) in high-, intermediate-, and low-risk patients.^1^^,^^2^ Patients with severe AS and severely calcified left main (LM) stenosis represent a special cohort with a high mortality risk, especially in the presence of compromised left ventricular function (LVF). The main determinant of their short-term outcomes is the occurrence of complications during high-risk percutaneous coronary interventions (HR-PCI) related to the use of high-pressure balloon inflations and aggressive plaque debulking techniques. In these patients, the risk of haemodynamic collapse during HR-PCI represents a major challenge.

Recently, intravascular lithotripsy (IVL) has proven to be safe and effective while minimizing plaque disruption and distal-embolization risks.^3^ Moreover, mechanical circulatory support (MCS) devices enable permanent peri-procedural haemodynamic stability.

## Timeline

**Table:** 

Case 1	
Day 0	Cardiac arrest at home and resuscitation with two electric shocks. Electrocardiogram (ECG): Anterior–lateral T-wave inversion in the emergency department. Two-dimensional echocardiogram (2D-echo): A low-flow/low-gradient aortic stenosis (AS) with ejection fraction (EF) of 23% was diagnosed. Severe hypokinesia of anterior and inferior walls. Severe right ventricular failure and pulmonary hypertension.
Day 1	Heavily calcified severe stenosis of left main (LM) stem; severe calcified proximal stenosis of left anterior descending artery (LAD) and right coronary artery (RCA) at angiography. Syntax Score (SS): 36.
Day 2	Heart team (HT) team agreed with high-risk percutaneous coronary intervention (HR-PCI).
Day 3	Intravascular lithotripsy (IVL) and stenting of LM stem, LAD and RCA under active veno-arterial extracorporeal membrane oxygenation (VA-ECMO) support. Patient was weaned from VA-ECMO in the cath lab at the end of percutaneous coronary interventions (PCIs).
Day 9	True severe low-flow/low-gradient AS confirmed at low-dose dobutamine-stress ECG (EF: 41%).
Day 11	Transcatheter aortic valve replacement (TAVR) successfully performed without mechanical circulatory support.
Day 13	Discharge home.

**Case 2**	

Day 0	Admission for worsening dyspnoea and rest angina. In-hospital 2D-echo: severe AS, EF of 26%.
Day 1	Subtotal heavily calcified LM stenosis of bifurcation, calcified stenosis of the proximal LAD, chronic total RCA occlusion, and left-to-right collateral filling at angiography. SS: 33.
Day2	HT agreed with a single-stage procedure of PCI and TAVR.
Day 3	VA-ECMO combined with intra-aortic balloon pump (IABP) activation. Treatment for chronic total RCA occlusion; IVL and culotte stenting of LM artery; and IVL and stenting of LAD. IABP interrupted at the end of PCIs. TAVR under active VA-ECMO support. Patient weaned from VA-ECMO immediately after intervention.
Day 7	Discharge home.

## Case presentation

### Case 1

A 78-year-old male ex-smoker was admitted after experiencing cardiac arrest and was resuscitated for sustained ventricular tachycardia with two electric shocks. Upon arrival at the emergency department, physical examination showed cold sweat, weak pulse, and a harsh aortic systolic murmur.

**Figure 1:**
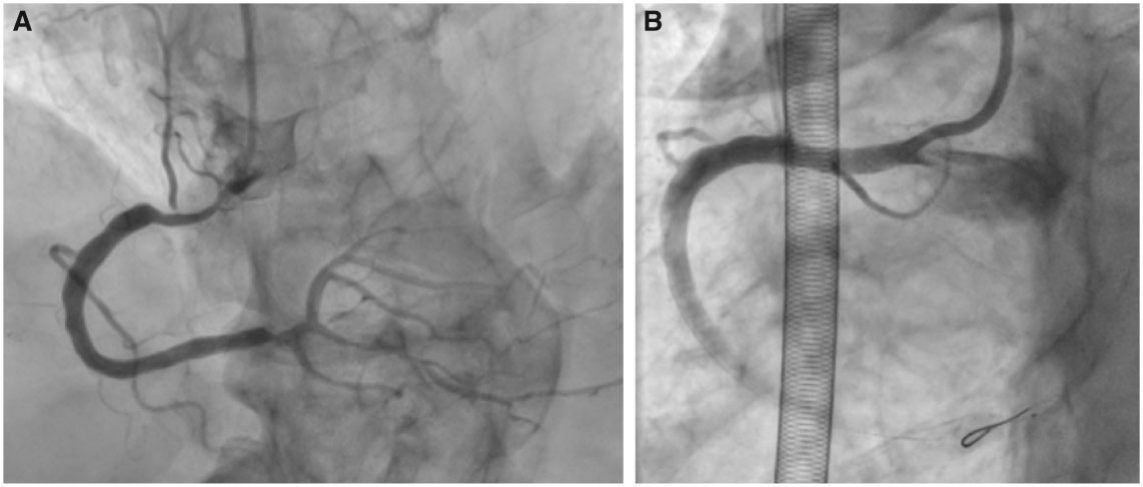
(*A*) Right coronary artery: diffuse critical narrowing. (*B*) Right coronary artery: final angiographic result.

He reported worsening dyspnoea and angina in the previous 2 weeks. His medical history consisted of moderate AS and severe chronic obstructive pulmonary disease (COPD) with FEV_1_ reduction of 40%. An electrocardiogram (ECG) showed anterior–lateral T-wave inversion. In the hospital, the two-dimensional echocardiogram (2D-echo) showed a severely calcified aortic valve [aortic valve area (AVA): 0.9cm^2^; indexed AVA: 0.67 cm^2^; peak velocity: 297 cm/s, mean pressure gradient: 25 mmHg; stroke volume index: 32 mL/m^2^] and a left ventricular ejection fraction (LVEF) of 23% (*Video 1*). The right ventricle appeared severely compromised, with pulmonary artery systolic pressure of 68 mmHg. Coronary angiography (CAG) revealed extensive calcification of both coronary arteries with multiple critical stenoses of the LM stem (*Video 2*), the left anterior descending artery (LAD) and the dominant right coronary artery (RCA; *Figure 1A*), yielding a Syntax Score (SS) of 36. The heart team (HT) discouraged surgery [logistic EuroSCORE II: 9.26%; Society of Thoracic Surgeons (STS) score: 4.12%], and agreed to offer percutaneous coronary intervention (PCI) at first. Rotational atherectomy was discouraged, considering the high risk of complications such as haemodynamic collapse, slow/no reflow and large dissection. Consequently, we selected IVL as a plaque debulking strategy. Considering the impaired biventricular contractility and the potential need for circulatory flow rate increase, circulatory veno-arterial extracorporeal membrane oxygenation (VA-ECMO) was chosen to supplement HR-PCI.

After ketamine sedation of the patient, we used two pre-closure devices (ProGlide; Abbott Cardiovascular, Clonmel, Ireland) on the left common femoral artery, inserted the cannulae percutaneously with ultrasound (US) guidance and activated VA-ECMO. Unfractionated heparin was used, with activated clotting time ranging from 180 to 250 s.

High-pressure pre-dilation (3.0/20-mm non-compliant balloon) of the focal LM stem stenosis was ineffective as balloon waist persisted. Therefore, intravascular ultrasound (IVUS) was performed, and multiple ‘short-shock’ pulses (separate balloon inflations, 5 pulses each) of IVL were applied with a 4.0/12-mm balloon inflated up to 4 ATM (up to 6 ATM if result is suboptimal).^4^ The balloon waist was no longer visible; therefore, we expanded a 3.5/22-mm drug-eluting coronary stent (DES) from the LM to the LAD. Furthermore, LAD stenosis was resolved by IVL, permitting a complete 3.0/18-mm DES expansion. High-pressure final optimization was obtained with excellent angiographic and IVUS results (*Video 2*). Finally, after suboptimal pre-dilation, IVL successfully pre-treated the RCA stenosis, allowing the implantation and optimization of a 3.5/28-mm DES (*Figure 1B*). IVUS confirmed full stent expansion.

The patient was weaned from VA-ECMO in the catheterization laboratory. On Day 7, the in-hospital 2D-echo showed that LVEF had risen to 41% (*Video 1*), and the mean aortic gradient was 38 mmHg. On Day 9, low-dose dobutamine-stress echocardiography (DSE) was performed to confirm true low-flow/low-gradient severe AS before proceeding with TAVR. On 10 µg/kg/min low-dose DSE, the mean aortic gradient increased to 45 mmHg, and AVA was 0.78 cm^2^, confirming a true severe low-flow/low-gradient AS. Two days later, a CoreValve Evolut-R 29 bioprosthesis (Medtronic, Dublin, Ireland) was implanted through right femoral access without any MCS (*Video 3*). Medical therapy at discharge was aspirin + ticagrelor, amiodarone, diuretics, and calcium antagonists. At 1-month follow-up, the patient was asymptomatic for dyspnoea and angina. At the 4-month 2D-echo evaluation, LVEF was 45%, and pulmonary artery systolic pressure of 50 mmHg.

### Case 2

**Figure 2:**
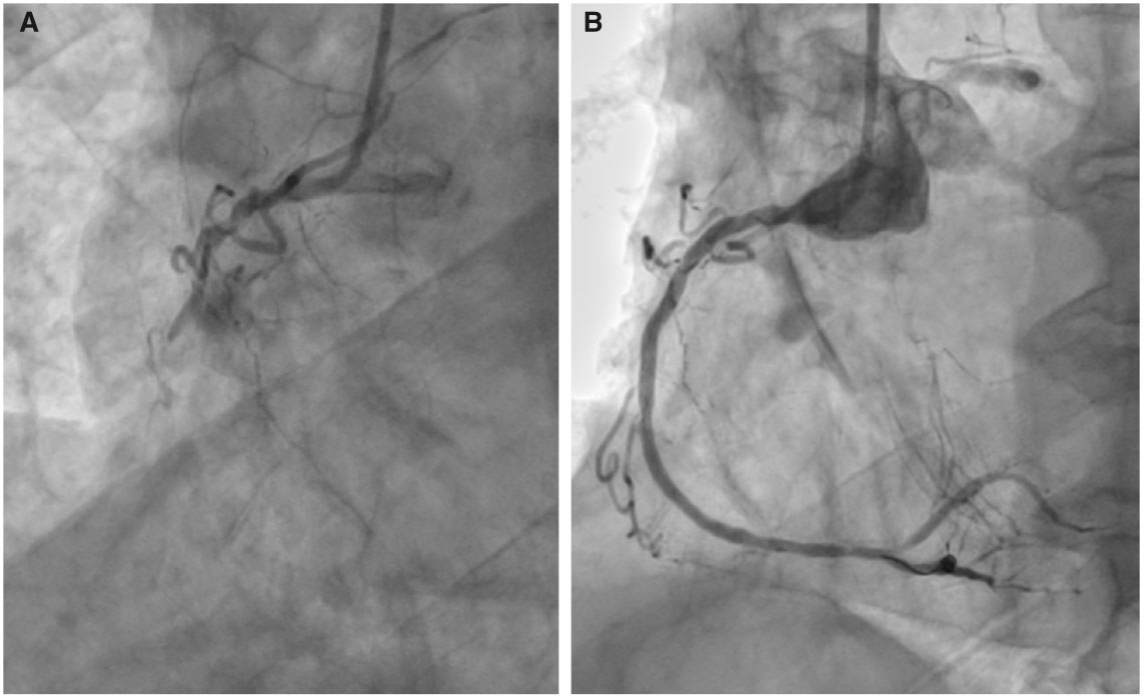
(*A*) Right coronary artery: chronic total occlusion. (*B*) Right coronary artery: final result.

A 79-year-old male smoker was admitted to our institution for worsening exertional dyspnoea over a 1-month period and rest angina within the previous few days. His physical examination revealed difficulty breathing, pulmonary basal crepitations, and a harsh aortic systolic murmur. Medical history consisted of severe hypertension and moderate COPD (FEV_1_ = 63%). In-hospital 2D-echo showed LVEF of 26% and severe AS (peak velocity: 480 cm/s; AVA: 0.6 cm^2^; maximum aortic gradient: 59 mmHg). CAG revealed severe and heavily calcified LM stenosis of the bifurcation (Supplementary material online, *Video S1*). Furthermore, calcified stenosis of the proximal LAD and a 10-mm long chronic total RCA occlusion were also seen (*Figure 2A*). SS was 33. Owing to patient’s age and operational risks (LEII: 9.63%; STS score: 6.15%), the HT decided on a single-stage, multi-vessel PCI and TAVR. Multi-slice computed tomography (MSCT) imaging confirmed valve and LM calcifications. Therefore, the HT opted for VA-ECMO + intra-aortic balloon pump (IABP) to support the coronary intervention. VA-ECMO was implanted via the left femoral artery by surgical exposure, because both femoral arteries were severely calcified. We percutaneously inserted the IABP via the right femoral artery. The RCA was treated first (*Figure 2B*), in order to reduce the global risk area during the subsequent LM PCI. Then, we performed IVL (2 cycles of 10 pulses each) on both the LAD and LM stenoses. The LM was treated via the double-stent ‘culotte’ technique (Supplementary material online, *Video S1*). After the coronary intervention, IABP was stopped, and TAVR began under active VA-ECMO support. We implanted a CoreValve Evolut-R 29 bioprosthesis (Medtronic, Dublin, Ireland; Supplementary material online, *Video S2*). The patient was weaned from VA-ECMO immediately after the intervention. Transthoracic ECG performed 2 days later showed an LVEF of 33%. Medical therapy at discharge was aspirin + ticagrelor, diuretics, and calcium antagonists. At 6-month follow-up, only mild effort dyspnoea was reported, and 2D-echo showed an LVEF of 48%.

## Discussion

We present two patients suffering from AS and calcified LM stenosis with severely impaired LVFs. In these patients, PCI + TAVR proved to be a viable alternative to high-risk surgery. However, this particular cohort of patients presents multiple challenges.

To assess AS severity in clinical decision-making, the gold standard is valve area measurement, along with flow rate, mean pressure gradient and ventricular function. The second case represented a patient with a true severe/high-gradient AS and valve area <1 cm^2^, irrespective of whether LVEF and flow rate were normal or reduced.^1^ Therefore, in agreement with current guidelines, both TAVR and PCI were undoubtedly recommended from the outset. Conversely, in the first case, a low-flow/low-gradient AS with reduced LVEF occurred. For this reason, low-dose DSE was recommended to distinguish truly severe from pseudo-severe AS. Thus, PCI was performed first, followed by DSE to confirm the severity of AS, and finally TAVR.

Most TAVR candidates have complex coronary artery disease. High-risk PCI performed before TAVR avoids several technical challenges and haemodynamic collapse, which can occur when significant proximal coronary segment stenosis has yet to be treated.

Further issues hinder the management of calcific LM stenosis. Rotational atherectomy combined with multiple and prolonged balloon inflations is always worrisome because of the high risk of complications.^3^ Conversely, IVL allows controlled fractures, minimizing plaque disruption and distal-embolization risk and thereby reducing the risk of acute LVF decompensation.^4^^,^^5^ The latter can be offset by the use of MCS, although no firm recommendations exist for or against MCS in stable patients undergoing complex HR-PCIs.^6^ These procedures can lead to prolonged periods of systemic and coronary hypoperfusion. The ultimate result can be myocardial depression and circulatory collapse, especially in patients with concomitant severe AS and compromised LVF. From a clinical standpoint, all MCS devices should have a place in high-risk PCIs, although they cannot be considered the standard of care in every procedure. Whether to use MCS depends on the benefit/risk ratio between the increase in procedural costs, length of surgery, and risk of vascular complications arising with their use; and the benefit derived from the protection of large at-risk myocardial areas during complex HR-PCI.

The results of extensive experiments conducted with VA-ECMO in high-risk PCI or TAVR report that the shorter the procedure time, the higher the probability the patient can be weaned off VA-ECMO. In addition, the prophylactic use of VA-ECMO implanted before PCI or TAVR increases bailout surgery survival chances.^7^ Both patients were informed of these facts, and they agreed to the surgeries.

Heavy vascular calcifications and peripheral instrumentation with large-bore cannulae such as Impella (Abiomed, Danvers, MA, USA) or VA-ECMO increase risks. In our patients, MSCT imaging showed that peripheral vessel size could allow large cannulae. Furthermore, VA-ECMO can be inserted using US guidance only, unlike other MCS devices, which require fluoroscopy. Finally, as closure devices see more widespread use, most VA-ECMO procedures could be fully percutaneous, and haemostasis could be immediately achieved.

A growing number of single-centre experiences support the role of VA-ECMO used electively during complex HR-PCI or TAVR.^8^ However, some detrimental side effects should be evaluated: VA-ECMO cannot provide adequate ventricular unloading, which inevitably increases myocardial oxygen consumption and myocardial work. Therefore, left ventricular venting assisted by IABP might be ultimately required to mitigate the aforementioned drawbacks. Furthermore, IABP adds some positive effects, such as an increase in coronary perfusion. A large meta-analysis^9^ and the nationwide Japanese inpatient database^10^ both reported that VA-ECMO + IABP significantly decreased in-hospital and 28-day mortality compared with VA-ECMO alone in patients undergoing HR-PCIs. In fact, aside from biventricular failure and pulmonary hypertension, other essential factors must be considered before the combined use of ECMO + IABP, such as the duration and complexity of the PCI and the expected ischaemic burden during it. If a high-risk PCI is expected, the risk related to the detrimental effect of left ventricular unloading during the use of VA-ECMO alone is concrete. For example, PCI of an LM lesion, especially when combined with significant RCA disease, can lead to massive ischaemia and ventricular decompensation. Moreover, complex bifurcation lesions of the distal LM stem might lead to ischaemia of longer duration. In the second patient, VA-ECMO + IABP were used because of the long-lasting intervention requiring complex double-stenting, with a high potential for life-threatening ischaemic complications. Conversely, when a simple single-stent strategy is presumed, VA-ECMO alone can be considered enough to support PCI.

Impella demonstrated its effectiveness in this setting^11^; however, we discarded its use for several reasons. First, a sufficient right ventricular performance is necessary to maintain left ventricular preload (*Table 1*). Second, the pump should be removed during TAVR. Third, Impella implantation can sometimes require a risky pre-dilation of the aortic valve before treating the coronary stenosis. This can precipitate acute or worsening aortic regurgitation or coronary artery embolization.

Finally, when a large ischaemic burden and a long-lasting, complex PCI are expected, VA-ECMO combined with IABP can overcome some of the side effects of VA-ECMO and limitations of the Impella heart pump.

## Conclusions

In the presented cases, IVL proved to be effective in managing heavily calcified LM coronary lesions, especially in the presence of an impaired LVF. In this setting, VA-ECMO was a valid MCS device to support high-risk multi-vessel PCIs and TAVR. Randomized studies are needed to evaluate the effect of VA-ECMO or VA-ECMO + IABP on the short-term outcomes of these high-risk patients.

## Lead author biography

**Figure:**
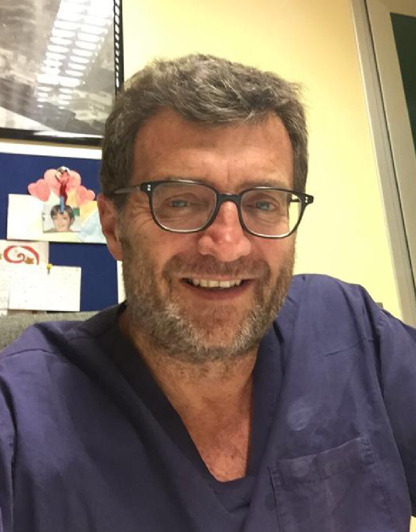


Dr Alfredo Marchese is the Chief of the Interventional Cardiology at Ospedale Santa Maria, in Bari, Italy. He has a prominent role in the executive board of the Italian Society of Interventional Cardiology. He has a particular expertise in the treatment of the left main coronary artery and of bifurcatiotion lesions. His research interests include optimizing antiplatelet therapy during elective CHIP-PCI and minimizing coronary access failure during TAVR procedures.

## Supplementary material

Supplementary material is available at *European Heart Journal - Case Reports* online.

**Slide sets:** A fully edited slide set detailing this case and suitable for local presentation is available online as supplementary data.

**Consent:** The authors confirm that written consent for submission and publication of this case report including images and associated text has been obtained from the patient in line with Committee on Publication Ethics (COPE) guidance.

**Conflict** **of interest:** None declared.

**Funding:** This work was supported by ‘Società Italiana di Cardiologia Interventistica-Gruppo Italiano di Studi Emodinamici’ (SICI-GISE).

**Table 1:** Mechanical cardiac support devices: pros and cons

Device	Pros	Cons
IABP	Very easy insertion and removal Readily available Low cost Reduces LV afterload	Modest support No support in case of arrhythmias LV support only
Impella	Direct LV unloading High support Efficacy independent of rhythm	Risk of vascular injury LV support only Significant cost
ECMO	Biventricular support ± oxygenator High support Full support in case of arrhythmias	Risk of vascular injury Increased LV afterload

## Supplementary Material

ytab498_Supplementary_Data
