# Supplementary material for: Mechanical circulatory support and intravascular lithotripsy in high-risk patients undergoing percutaneous coronary intervention and transcatheter aortic valve replacement: a case series
Source: Eur Heart J Case Rep. 2021 Dec 7;5(12):ytab498. doi: 10.1093/ehjcr/ytab498 (PMC8759477; doi:10.1093/ehjcr/ytab498)
Supplement: ytab498_Supplementary_Data [file ytab498_Supplementary_Data.zip › Slide-Set.pptx]

## Slide 1
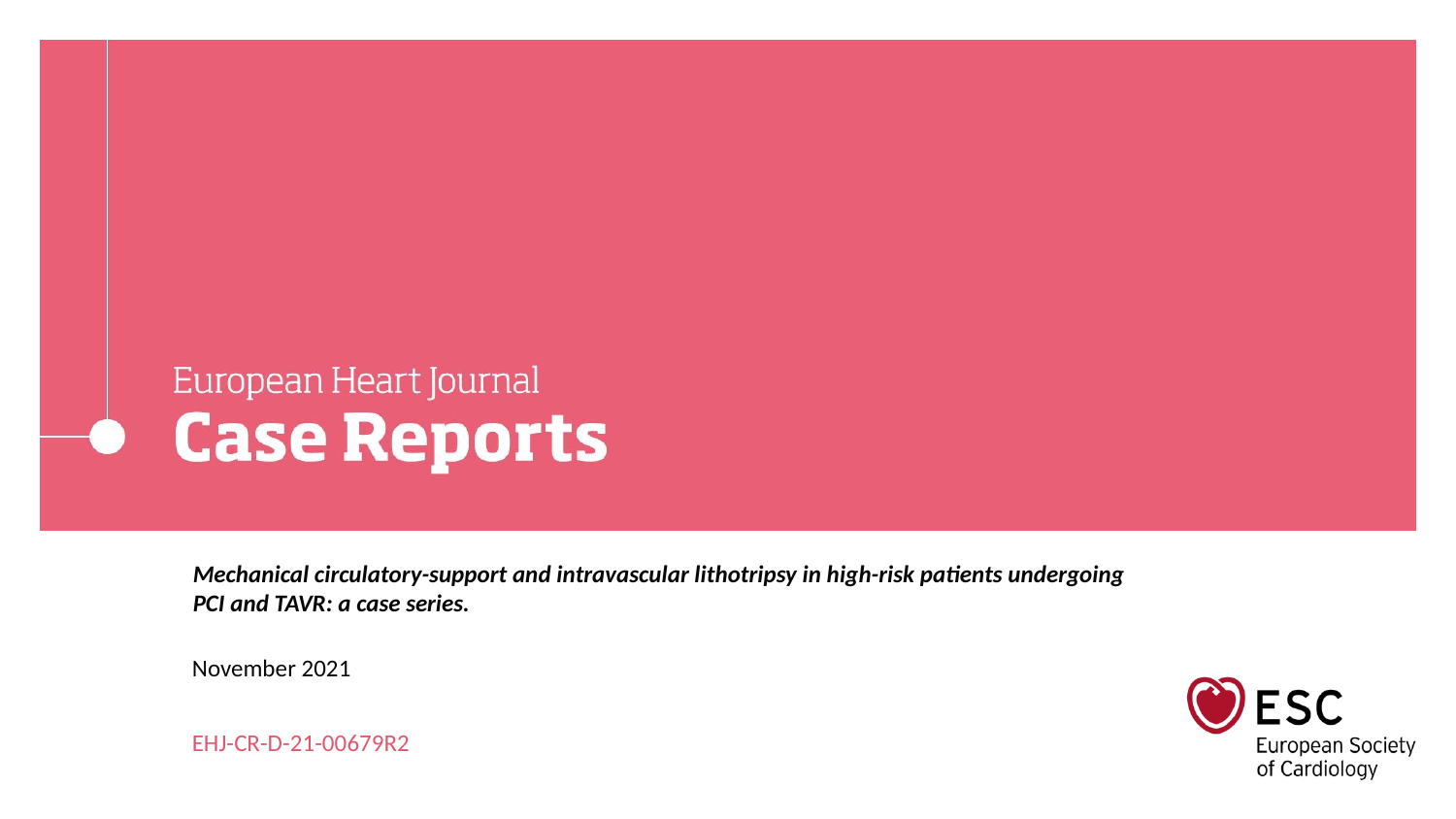

# Mechanical circulatory-support and intravascular lithotripsy in high-risk patients undergoing PCI and TAVR: a case series.
November 2021
EHJ-CR-D-21-00679R2

## Slide 2
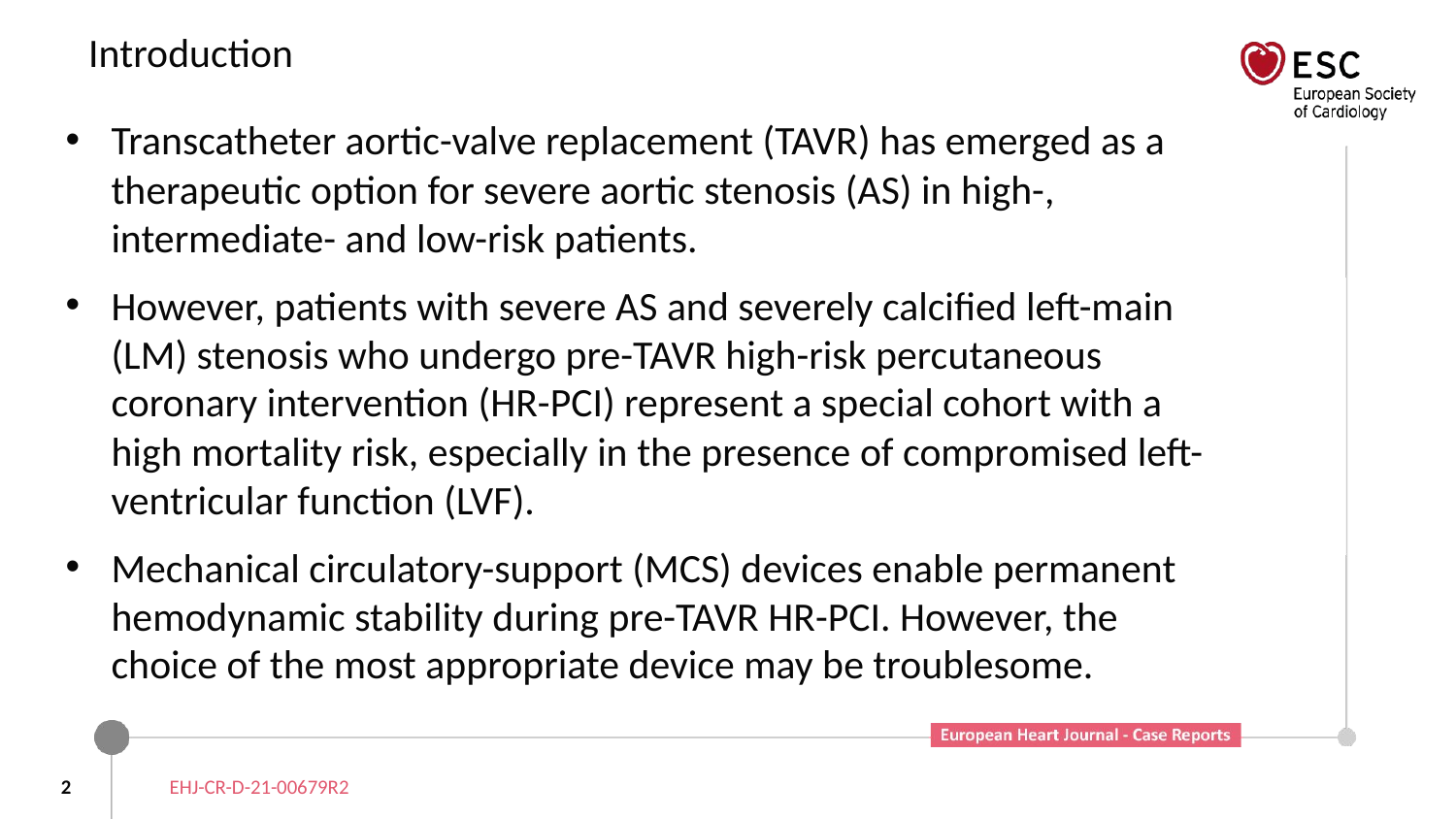

# Introduction
Transcatheter aortic-valve replacement (TAVR) has emerged as a therapeutic option for severe aortic stenosis (AS) in high-, intermediate- and low-risk patients.
However, patients with severe AS and severely calcified left-main (LM) stenosis who undergo pre-TAVR high-risk percutaneous coronary intervention (HR-PCI) represent a special cohort with a high mortality risk, especially in the presence of compromised left-ventricular function (LVF).
Mechanical circulatory-support (MCS) devices enable permanent hemodynamic stability during pre-TAVR HR-PCI. However, the choice of the most appropriate device may be troublesome.
2
EHJ-CR-D-21-00679R2

## Slide 3
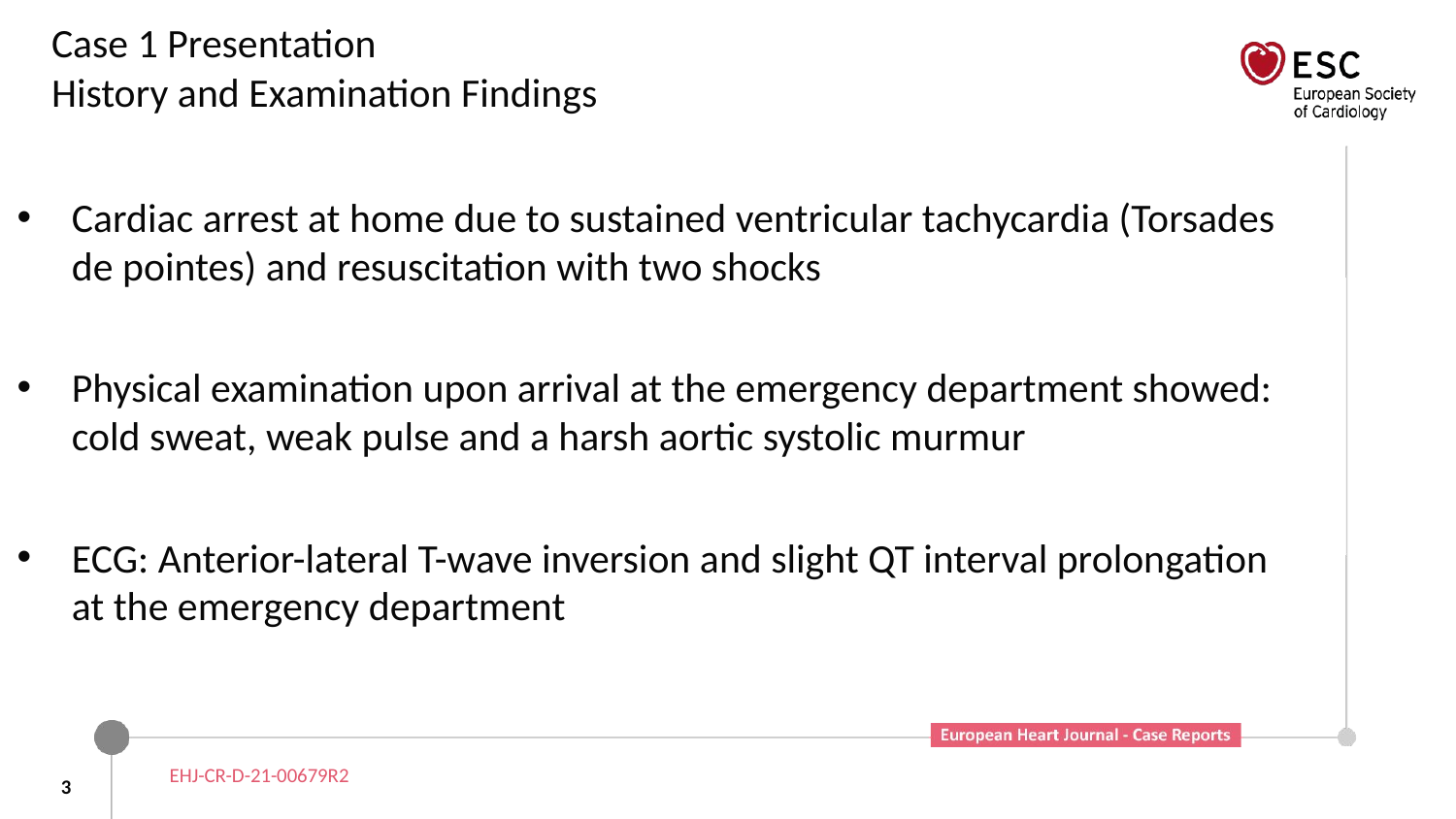

# Case 1 Presentation History and Examination Findings
Cardiac arrest at home due to sustained ventricular tachycardia (Torsades de pointes) and resuscitation with two shocks
Physical examination upon arrival at the emergency department showed: cold sweat, weak pulse and a harsh aortic systolic murmur
ECG: Anterior-lateral T-wave inversion and slight QT interval prolongation at the emergency department
3
EHJ-CR-D-21-00679R2

## Slide 4
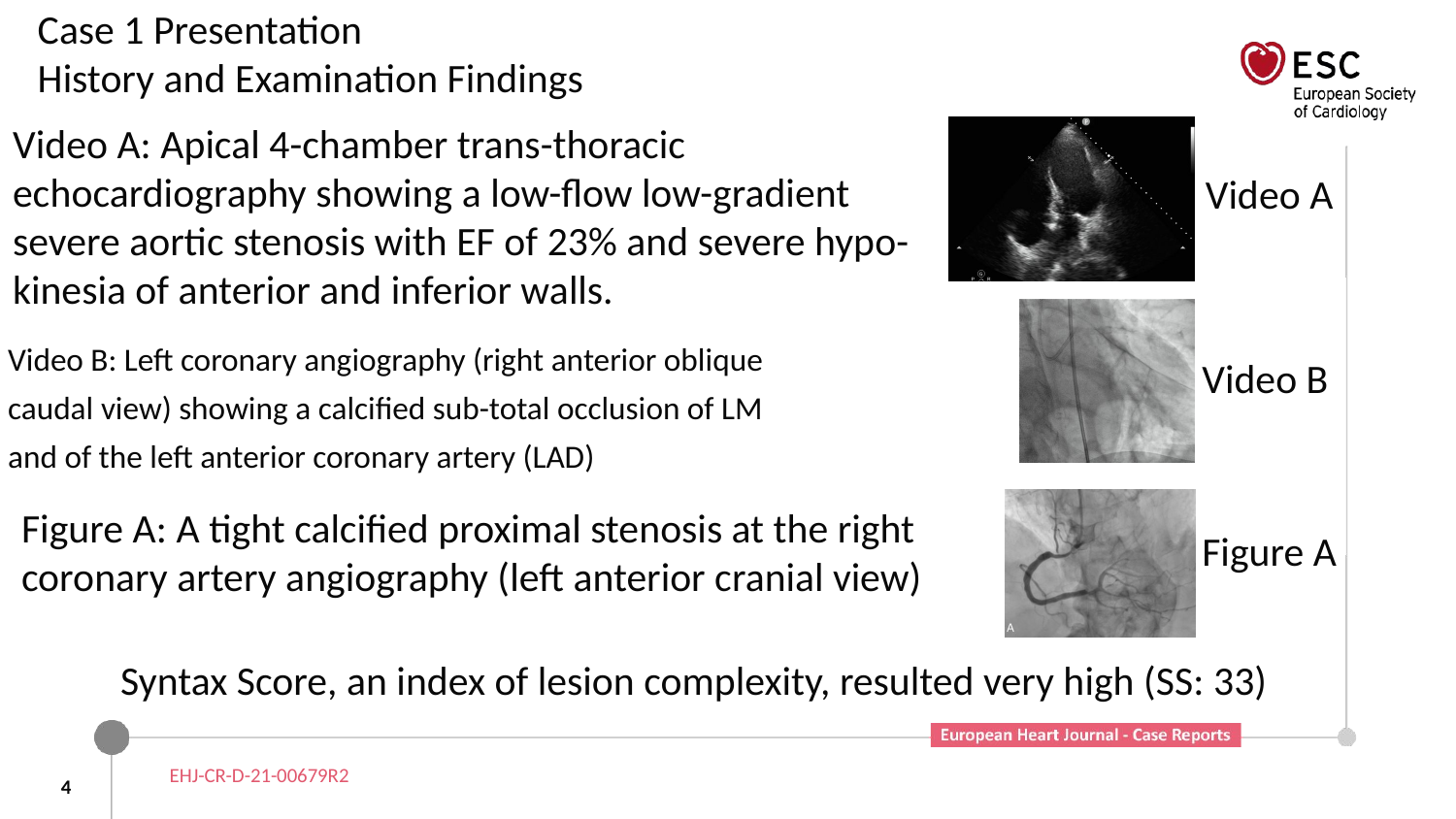

# Case 1 Presentation History and Examination Findings
Video A: Apical 4-chamber trans-thoracic echocardiography showing a low-flow low-gradient severe aortic stenosis with EF of 23% and severe hypo-kinesia of anterior and inferior walls.
Video A
Video B: Left coronary angiography (right anterior oblique
caudal view) showing a calcified sub-total occlusion of LM
and of the left anterior coronary artery (LAD)
Video B
Figure A: A tight calcified proximal stenosis at the right
coronary artery angiography (left anterior cranial view)
Figure A
Syntax Score, an index of lesion complexity, resulted very high (SS: 33)
4
EHJ-CR-D-21-00679R2

## Slide 5
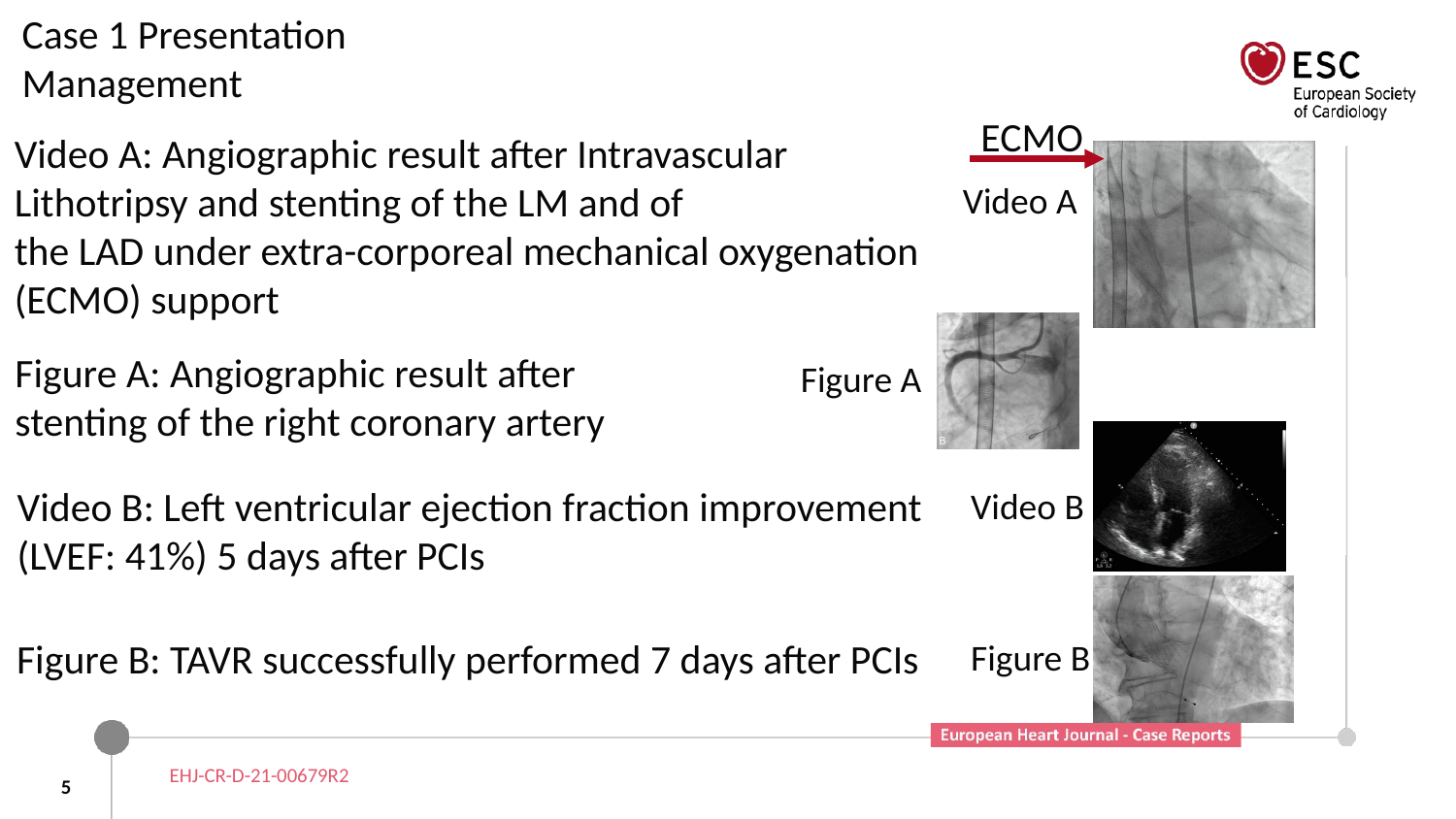

# Case 1 Presentation Management
ECMO
Video A: Angiographic result after Intravascular Lithotripsy and stenting of the LM and of
the LAD under extra-corporeal mechanical oxygenation (ECMO) support
Video A
Figure A: Angiographic result after
stenting of the right coronary artery
Figure A
Video B: Left ventricular ejection fraction improvement
(LVEF: 41%) 5 days after PCIs
Video B
Figure B: TAVR successfully performed 7 days after PCIs
Figure B
5
EHJ-CR-D-21-00679R2

## Slide 6
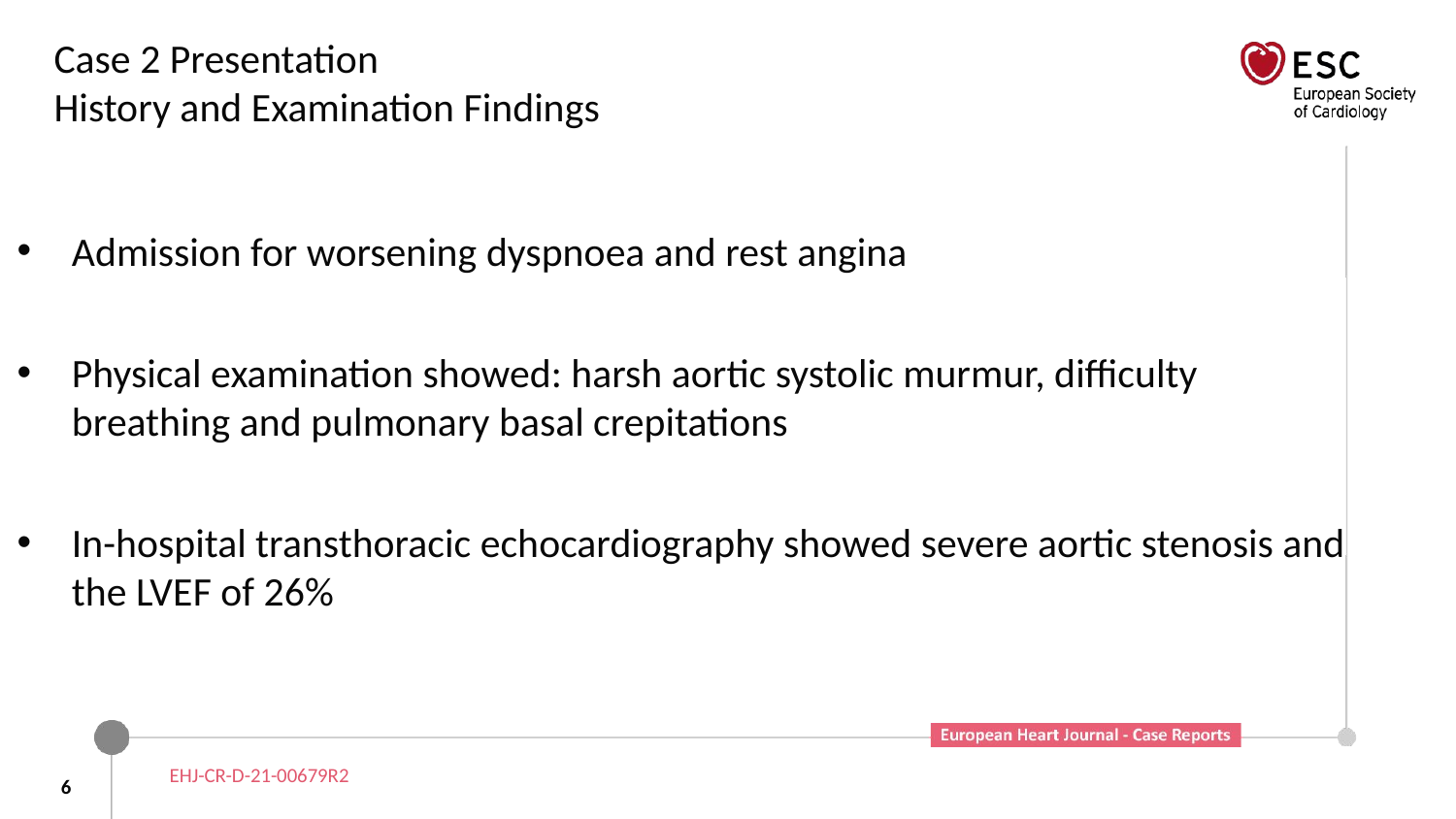

# Case 2 Presentation History and Examination Findings
Admission for worsening dyspnoea and rest angina
Physical examination showed: harsh aortic systolic murmur, difficulty breathing and pulmonary basal crepitations
In-hospital transthoracic echocardiography showed severe aortic stenosis and the LVEF of 26%
6
EHJ-CR-D-21-00679R2

## Slide 7
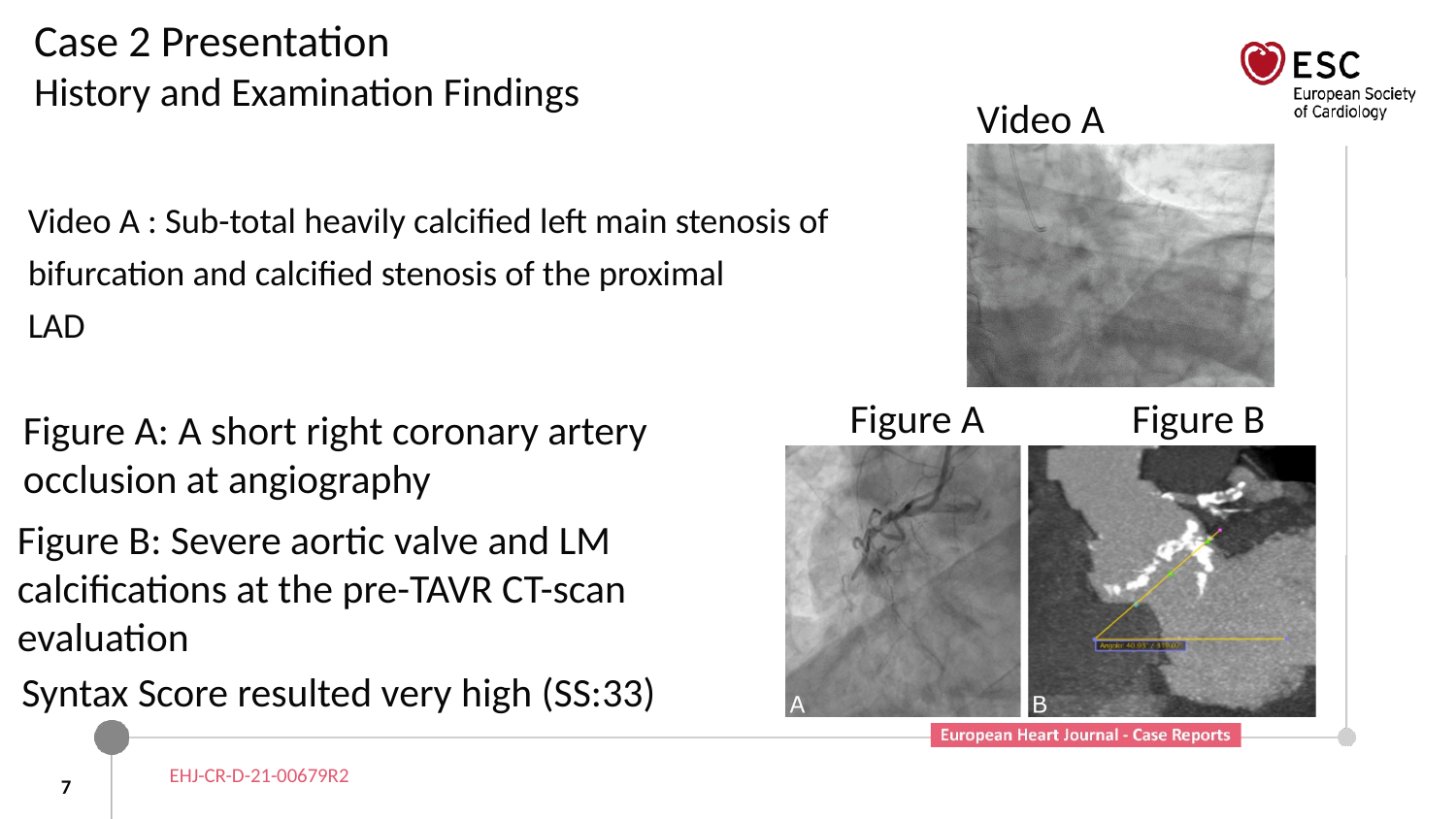

# Case 2 Presentation History and Examination Findings
Video A
Video A : Sub-total heavily calcified left main stenosis of
bifurcation and calcified stenosis of the proximal
LAD
Figure A Figure B
Figure A: A short right coronary artery
occlusion at angiography
Figure B: Severe aortic valve and LM
calcifications at the pre-TAVR CT-scan evaluation
Syntax Score resulted very high (SS:33)
7
EHJ-CR-D-21-00679R2

## Slide 8
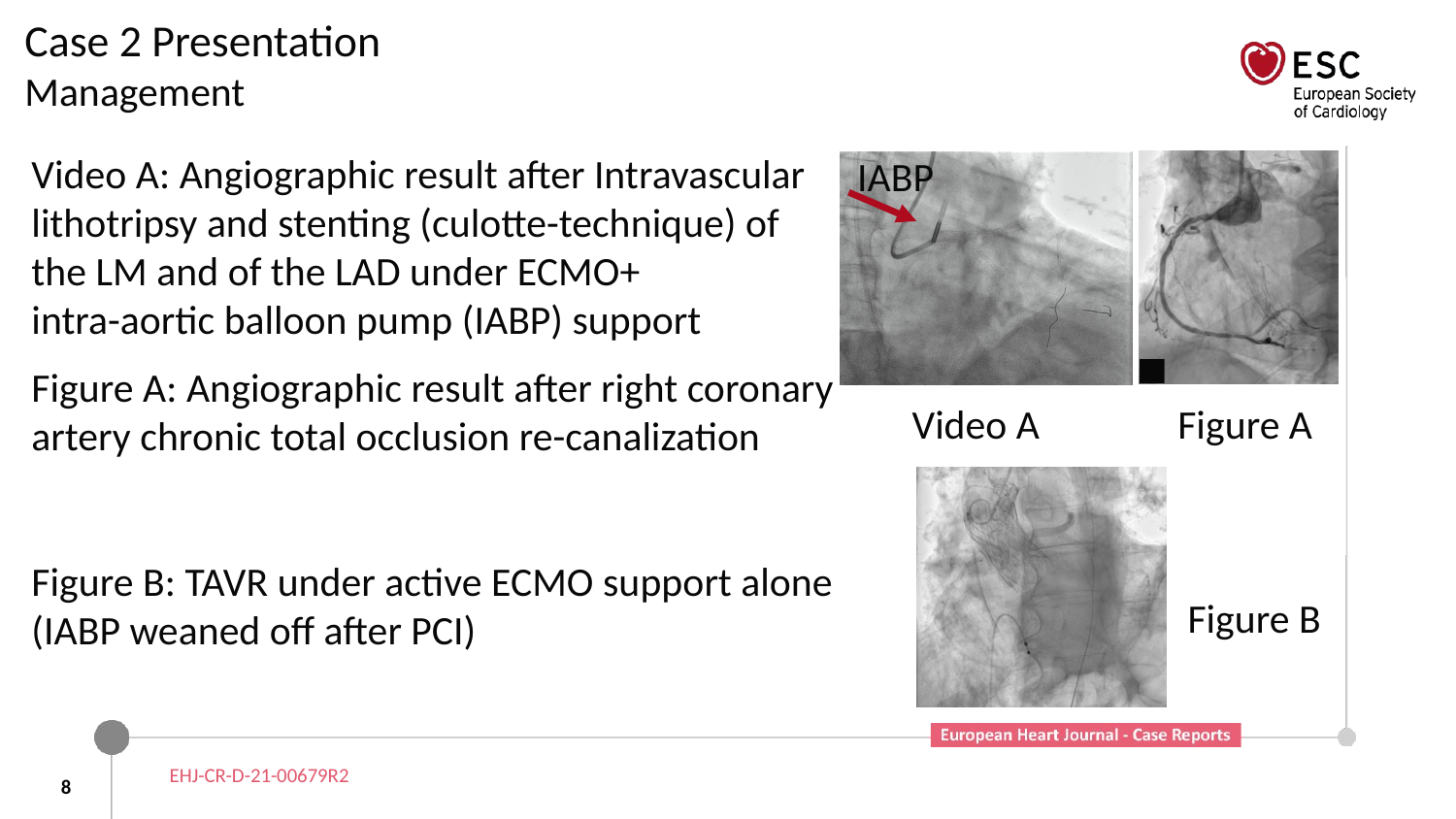

# Case 2 Presentation Management
Video A: Angiographic result after Intravascular lithotripsy and stenting (culotte-technique) of the LM and of the LAD under ECMO+
intra-aortic balloon pump (IABP) support
Figure A: Angiographic result after right coronary artery chronic total occlusion re-canalization
Figure B: TAVR under active ECMO support alone (IABP weaned off after PCI)
IABP
 Video A Figure A
Figure B
8
EHJ-CR-D-21-00679R2

## Slide 9
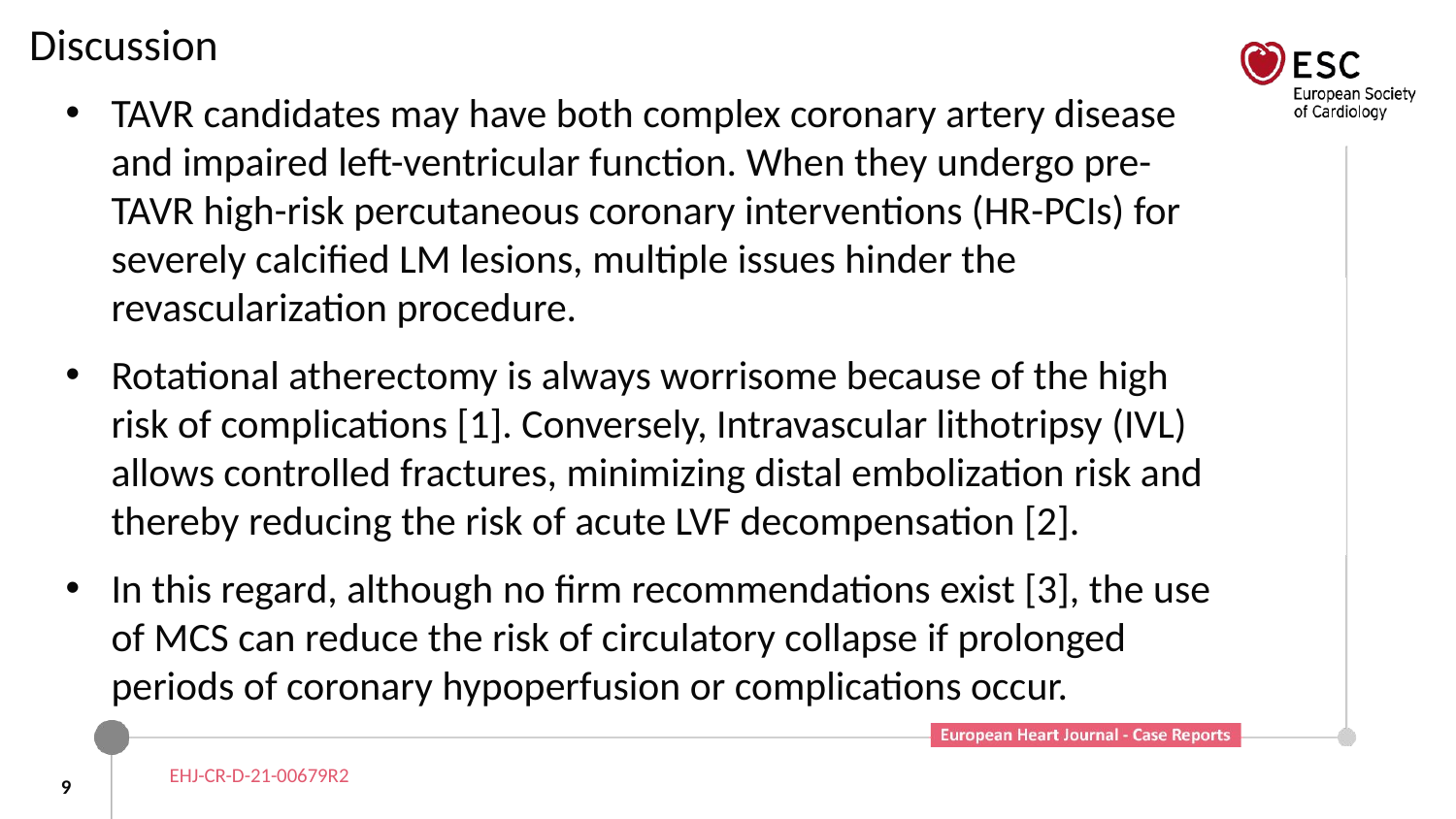

# Discussion
TAVR candidates may have both complex coronary artery disease and impaired left-ventricular function. When they undergo pre-TAVR high-risk percutaneous coronary interventions (HR-PCIs) for severely calcified LM lesions, multiple issues hinder the revascularization procedure.
Rotational atherectomy is always worrisome because of the high risk of complications [1]. Conversely, Intravascular lithotripsy (IVL) allows controlled fractures, minimizing distal embolization risk and thereby reducing the risk of acute LVF decompensation [2].
In this regard, although no firm recommendations exist [3], the use of MCS can reduce the risk of circulatory collapse if prolonged periods of coronary hypoperfusion or complications occur.
9
EHJ-CR-D-21-00679R2

## Slide 10
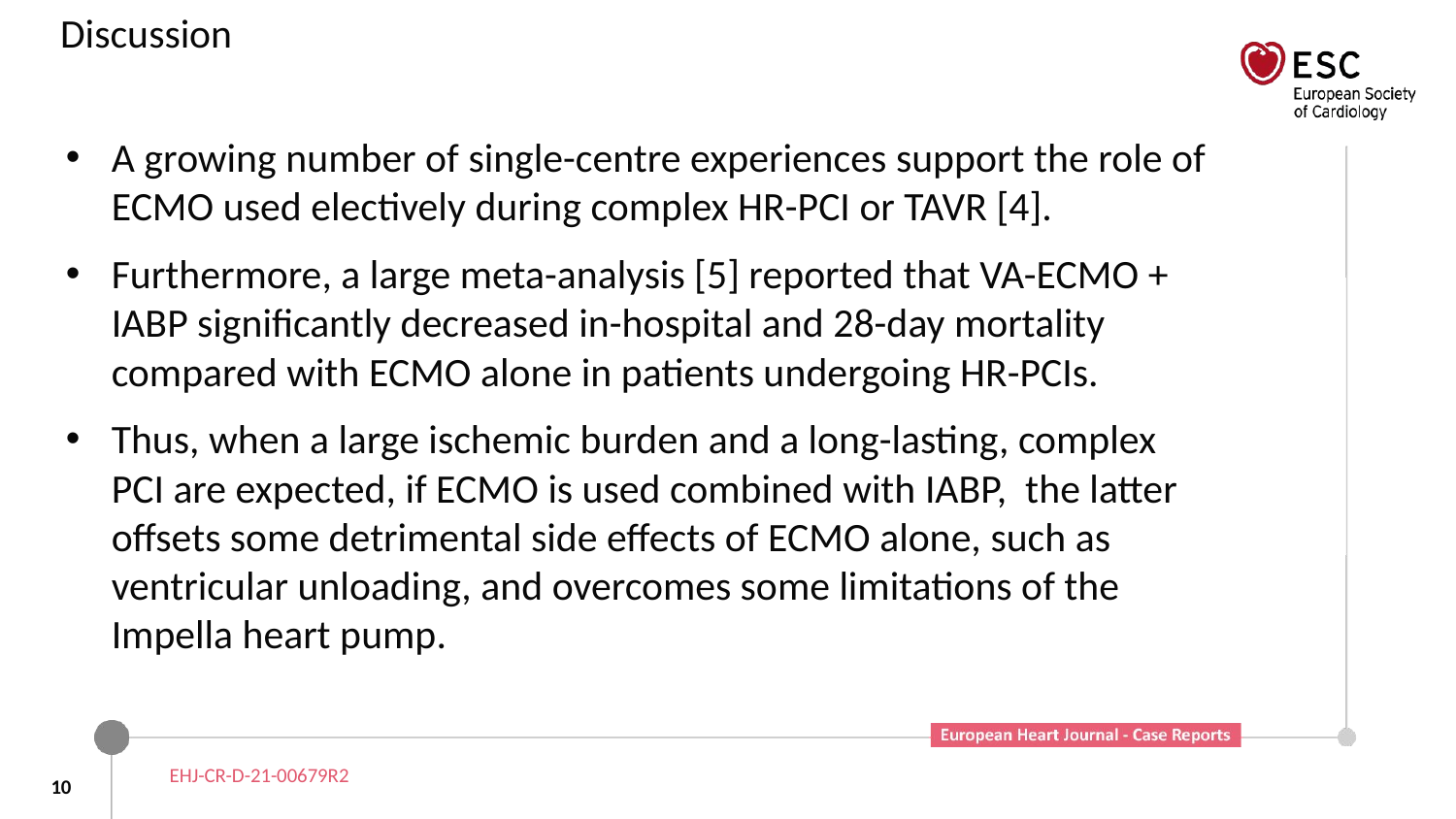

# Discussion
A growing number of single-centre experiences support the role of ECMO used electively during complex HR-PCI or TAVR [4].
Furthermore, a large meta-analysis [5] reported that VA-ECMO + IABP significantly decreased in-hospital and 28-day mortality compared with ECMO alone in patients undergoing HR-PCIs.
Thus, when a large ischemic burden and a long-lasting, complex PCI are expected, if ECMO is used combined with IABP, the latter offsets some detrimental side effects of ECMO alone, such as ventricular unloading, and overcomes some limitations of the Impella heart pump.
10
EHJ-CR-D-21-00679R2

## Slide 11
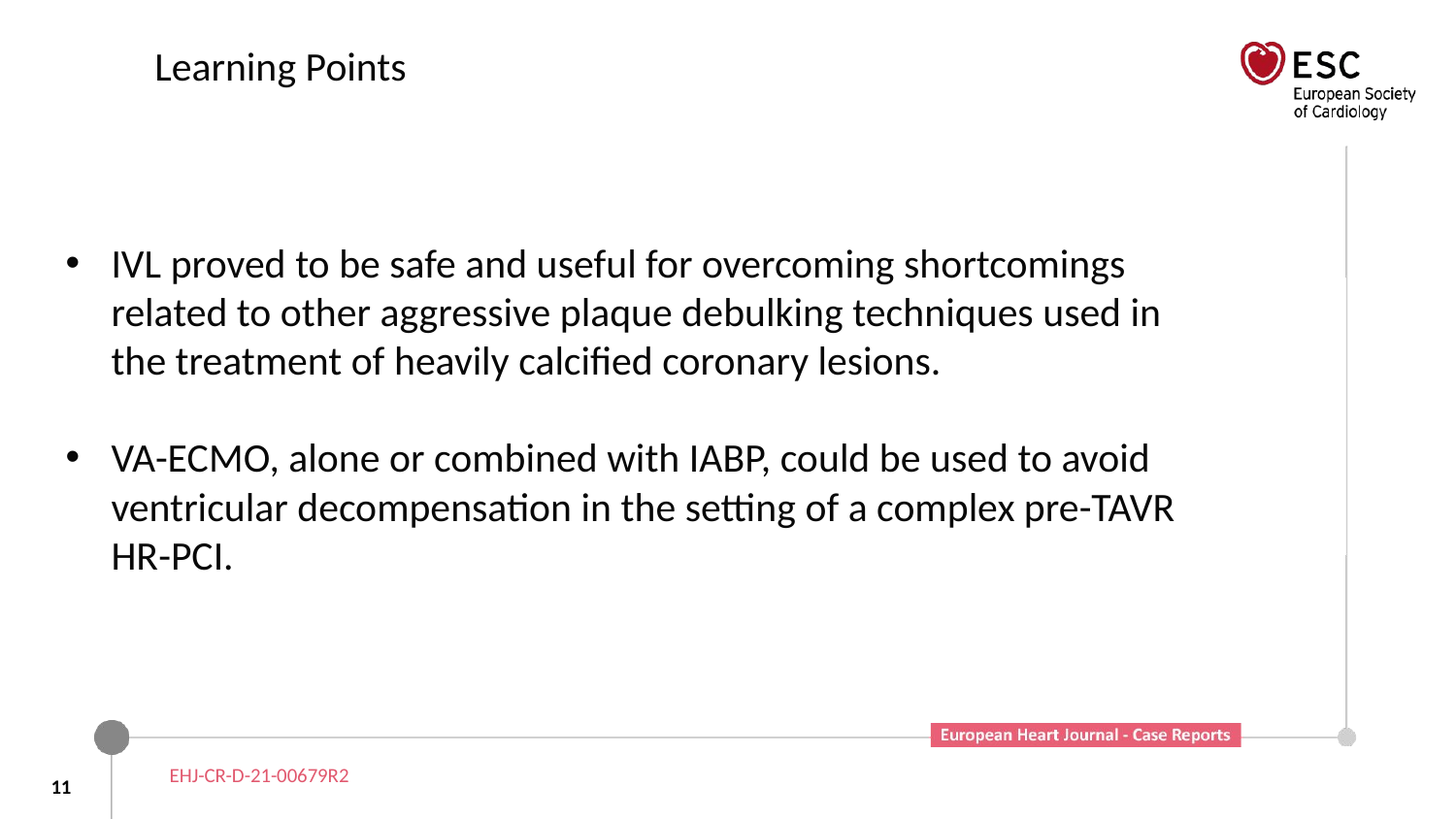

# Learning Points
IVL proved to be safe and useful for overcoming shortcomings related to other aggressive plaque debulking techniques used in the treatment of heavily calcified coronary lesions.
VA-ECMO, alone or combined with IABP, could be used to avoid ventricular decompensation in the setting of a complex pre-TAVR HR-PCI.
11
EHJ-CR-D-21-00679R2

## Slide 12
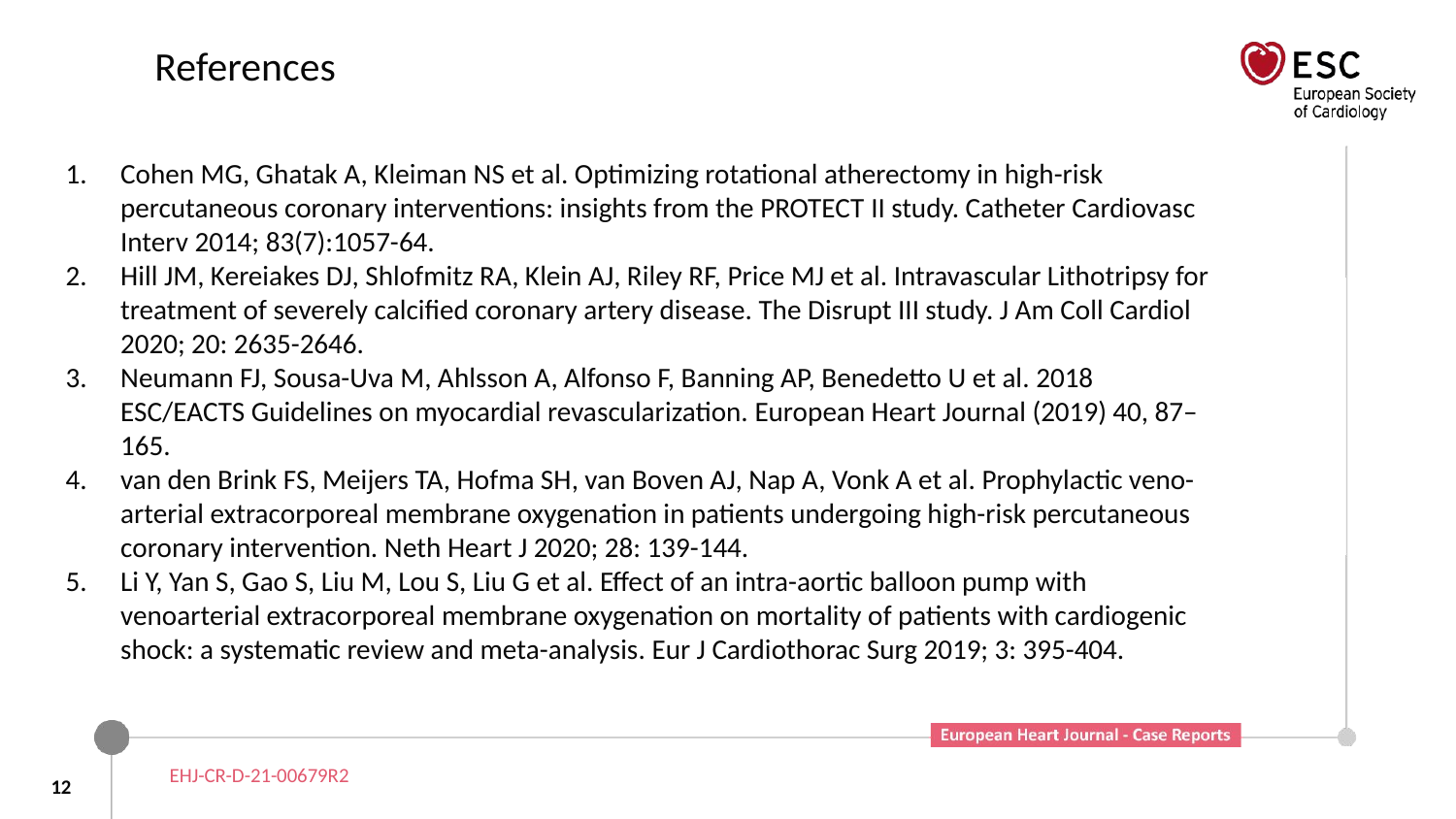

# References
Cohen MG, Ghatak A, Kleiman NS et al. Optimizing rotational atherectomy in high-risk percutaneous coronary interventions: insights from the PROTECT ΙΙ study. Catheter Cardiovasc Interv 2014; 83(7):1057-64.
Hill JM, Kereiakes DJ, Shlofmitz RA, Klein AJ, Riley RF, Price MJ et al. Intravascular Lithotripsy for treatment of severely calcified coronary artery disease. The Disrupt III study. J Am Coll Cardiol 2020; 20: 2635-2646.
Neumann FJ, Sousa-Uva M, Ahlsson A, Alfonso F, Banning AP, Benedetto U et al. 2018 ESC/EACTS Guidelines on myocardial revascularization. European Heart Journal (2019) 40, 87–165.
van den Brink FS, Meijers TA, Hofma SH, van Boven AJ, Nap A, Vonk A et al. Prophylactic veno-arterial extracorporeal membrane oxygenation in patients undergoing high-risk percutaneous coronary intervention. Neth Heart J 2020; 28: 139-144.
Li Y, Yan S, Gao S, Liu M, Lou S, Liu G et al. Effect of an intra-aortic balloon pump with venoarterial extracorporeal membrane oxygenation on mortality of patients with cardiogenic shock: a systematic review and meta-analysis. Eur J Cardiothorac Surg 2019; 3: 395-404.
12
EHJ-CR-D-21-00679R2
